# In vivo evaluation of the early events associated with liver metastasis of circulating cancer cells

**DOI:** 10.1054/bjoc.2001.1911

**Published:** 2001-08

**Authors:** L Ding, M Sunamura, T Kodama, J Yamauchi, D G Duda, H Shimamura, K Shibuya, K Takeda, S Matsuno

**Affiliations:** First Department of Surgery, Tohoku University School of Medicine, Seiryo-cho 1-1, Aoba-ku, Sendai, 980-8574, Japan

**Keywords:** circulating tumour cells, liver microcirculation, P-selectin, knockout mouse, intravital microscopy

## Abstract

The mechanism of metastasis formation remains still largely unknown. Many studies underline the importance and complexity of the initial arrest of the circulating tumour cells in the target organ, a key stage in metastasis occurrence. In our study, we evaluated by visual means the metastasis formation using an in vivo microscopy system in a murine model. Moreover, we investigated the involvement of P-selectin in these processes using immunohistochemistry and P-selectin knockout mice. The present study offers direct evidence of distinct pathways for tumour metastasis formation by a lymphoma cell – EL-4 and a solid tumour cell – C26. Off-line analysis of the images and histological data confirmed that mechanical entrapment of the solid tumour cell, which had a bigger diameter than that of the liver sinusoids, promoted metastasis without any detectable involvement of adhesion molecules. On the other hand, we observed that lymphoma cells, in spite of their smaller diameter as compared to the sinusoids, promoted liver metastasis as well, but with the essential participation in their arrest of P-selectin, indicating an adhesion molecule-mediated pathway. © 2001 Cancer Research Campaign http://www.bjcancer.com


*British Journal of Cancer* (2001) **85**(3), 431–438
© 2001 Cancer Research Campaign
doi: 10.1054/ bjoc.2001.1911, available online at http://www.idealibrary.com on IDEAL®

http://www.bjcancer.com

### In vivo evaluation of the early events associated with liver metastasis of circulating cancer cells

**L Ding, M Sunamura, T Kodama, J Yamauchi, DG Duda, H Shimamura, K Shibuya, K Takeda and S Matsuno**

First Department of Surgery, Tohoku University School of Medicine, Seiryo-cho 1-1, Aoba-ku, 980-8574 Sendai, Japan

**Summary** The mechanism of metastasis formation remains still largely unknown. Many studies underline the importance and complexity of the initial arrest of the circulating tumour cells in the target organ, a key stage in metastasis occurrence. In our study, we evaluated by visual means the metastasis formation using an in vivo microscopy system in a murine model. Moreover, we investigated the involvement of P-selectin in these processes using immunohistochemistry and P-selectin knockout mice. The present study offers direct evidence of distinct pathways for tumour metastasis formation by a lymphoma cell – EL-4 and a solid tumour cell – C26. Off-line analysis of the images and histological data confirmed that mechanical entrapment of the solid tumour cell, which had a bigger diameter than that of the liver sinusoids, promoted metastasis without any detectable involvement of adhesion molecules. On the other hand, we observed that lymphoma cells, in spite of their smaller diameter as compared to the sinusoids, promoted liver metastasis as well, but with the essential participation in their arrest of P-selectin, indicating an adhesion molecule-mediated pathway. © 2001 Cancer Research Campaign http://www.bjcancer.com

**Keywords**: circulating tumour cells; liver microcirculation; P-selectin; knockout mouse; intravital microscopy

Current understanding describes metastasis of malignant tumours as a multistage process involving the detachment of tumour cells from the primary site followed by their entry into the cardiovascular system and evasion of the immune surveillance. Subsequently, tumour cells or multicellular emboli are arrested in target organs, and finally, the extravasation and proliferation of these cells results in the formation of new tumour colonies at distant sites (Tarin and Matsumira, 1994). These events are characterized by complex interactions between the cancer cells and the endothelium involving the expression of integrins, matrix metalloproteinases, selectins and their ligands, cadherins, immunoglobulins and other uncharacterized factors (Orr et al, 2000).

The arrest of circulating cancer cells is an essential step for the success of metastasis. Several lines of evidence have been put forward to explain the mechanism of arresting. Some researchers consider that since most cancer cells are very large in size as compared to capillaries, cell arrest will occur in the organ that is first encountered by tumour cells after leaving the primary site. In other words, arrest is probably due to a non-specific entrapment of tumour cells in the microvasculature (Sugarbaker, 1981; Barbera-Guillem et al, 1989; Morris et al, 1993). On the other hand, it has also been reported that certain types of cancer cells preferentially adhere to the endothelium of selected organs. This process is presumed to be mediated by specific interactions between inducible adhesion molecules and their ligands (Pauli et al, 1990; Wang et al, 1999; Zetter, 1993).

P-selectin, E-selectin and their associated carbohydrate ligands, sialyl Lewis a ($sLe^a$) and sialyl Lewis x ($sLe^x$), are adhesion molecules that promote leukocyte rolling on activated endothelial cells (Bevilacqua and Nelson, 1993). P-selectin and E-selectin are expressed in many organs in humans, such as the lung, liver, and kidney. P-selectin and E-selectin are also expressed in rodents upon stimulation with histamine, $H_2O_2$ or certain cytokines. Recent studies indicated that P-selectin, E-selectin and their ligands are closely associated with metastasis (Aigner et al, 1998). In this sense, supporting evidence has been offered by the fact that the level of expression of selectins, $sLe^a$ and $sLe^x$ increases in several types of malignant tissues and correlates with a poor prognosis (Nakamori et al, 1993). In vitro, the expression of P-selectin and E-selectin mediates the adherence of cancer cells to activated endothelial cells (Lauri et al, 1991; Zhu et al, 1991). Of particular interest, cancer cell lines with higher potential to metastasize exhibited stronger adhesion capability (Izumi et al, 1995), while organ-specific expression of E-selectin in transgenic mouse re-directed the site of organ-specific cancer metastasis (Biancone et al, 1996). However, the idea of a dominant role for selectins is controversial (Fox-Robichaud and Kubes, 2000) and there has not been any direct in vivo proof of their involvement in the tumour metastasis process.

The intravital microscopy system used in our study has the advantage to allow in vivo observation of organs and has been successfully applied in previous tumour metastasis studies (Kan et al, 1993; Chambers et al, 1995; Scherbarth and Orr, 1997; Chambers, 1999). The aim of this study was to investigate the role of P-selectin in the arrest of cancer cells using the intravital microscopic system and P-selectin knockout mice by comparing the interactions occurring in 2 different murine models, a lymphoma and a solid tumour.

*Received 20 March 2000*
*Revised 5 March 2001*
*Accepted 7 March 2001*

*Correspondence to:* M Sunamura

### MATERIALS AND METHODS

### Animals

Inbred BALB/c mice, 6–8 weeks old were purchased from CLEA Inc (Tokyo, Japan), and C57BL/6 mice, 6–8 weeks old were purchased from Funabashi Farm Inc (Sendai, Japan). P-selectin knockout mice (B6-129-SelP), male, 21 weeks, were purchased from Jackson Laboratory (Boston, MA). 21 week old normal wild-type C57BL/6 mice were used as control in the P-selectin deficient mice experiment. All mice were housed at the Institute of Experimental Animals, Tohoku University, and used in experimental settings that met the standards required by UKCCCR Guidelines.

### Cells and labelling

As stated previously, this study investigated the hepatic metastasis formation in a murine model using 2 cell lines: C26 colon adenocarcinoma originating from BALB/c mouse and EL-4 lymphoma originating from C57BL/6 black mouse. Both cell lines were cultured in RPMI-1640 medium supplemented with 10% fetal calf serum and maintained in a humidified incubator with 5% $CO_2$ atmosphere. Subconfluent C26 cells were detached using 0.25% trypsin and 0.02% EDTA PBS solution. EL-4 cells were detached by flushing the bottom of flasks with culture medium. Tumour cell labelling was performed using PKH-26 C Kit (Zynaxis Cell Science Inc, Malvern, PA). PKH-26 is a fluorescent dye that selectively binds to the cell lipid bilayer and is used for the observation of cellular adhesion and migration – applications for which its low cytotoxicity and high resistance to intercellular transfer make it ideally suited (Horan et al, 1989). Briefly, the staining process was performed as follows: cancer cells were harvested, then washed with serum-free RPMI-1640; one $\times 10^7$ C26 or $2 \times 10^7$ EL-4 cells were mixed with a 50-fold dilution of PKH-26 stock at room temperature for 5 minutes. The staining was terminated by adding 10% FCS and the cells were washed with 10% FCS-supplemented RPMI-1640 and then suspended in PBS for in vivo experiments. The percentage of living cells exceeded 90% following PKH-26 staining, as assessed by the trypan blue exclusion method.

### Monoclonal antibodies

We used purified rat anti-mouse P-selectin antibody (anti-CD62P) and anti E-selectin antibody (anti-CD62E) (PharMingen, Inc, San Diego, CA), which are neutralizing monoclonal antibodies. Mouse anti-mouse $sLe^a$ antibody (2D3) and anti-$sLe^x$ antibody (2H5) were kindly supplied by Dr Kannagi from the Department of Experimental Pathology, Aichi Cancer Research Institute, Nagoya, Japan.

### Flow cytometry

EL-4 and C26 cells ($1 \times 10^6$/sample) were individually incubated with anti-CD62P, anti-CD62E, 2D3 or 2H5 for 30 minutes. They were then washed and treated with FITC-labelled second antibodies (anti-rat-IgG for anti-CD62P and anti-CD62E incubated cells; anti-mouse-IgM for 2D3 and 2H5 incubated cells). After the final washing, the cells were resuspended and the fluorescent signals were collected and analysed using a FACScan cytometer (Becton Dickinson, Inc, San Jose, CA) operated by CELLQuest software (Becton Dickinson, Inc, San Jose, CA). Cells in control groups were treated only with the second antibodies.

### Immunohistochemical staining

The livers from C57BL/6 mice were harvested 30 minutes after the laparotomy. The specimens were fixed in phospho-lysine-paraformaldehyde (PLP) solution at 4°C over night, and then treated with a series of sucrose solutions in PBS, embedded in OCT compound and stored at –80°C. 5-µm-thick sections were cut and the immunohistochemical staining for P-selectin was performed using anti-CD62P monoclonal antibody and an ABC kit (Vector Laboratories, Burlingame, CA).

### Intravital microscopic analysis

Inverted microscope (DIAPHOT 300, Nikon, Inc, Tokyo, Japan) was designed for epi-illuminational fluorescence observation. The microscope was installed with an FITC filter (Nikon, Inc, Tokyo, Japan) and a triple band filter for DAPI, FITC and rhodamine (Nikon, Inc, Tokyo, Japan). Colon 26 cells for BALB/c mice and EL-4 cells for C57BL/6 mice were used to establish hepatic metastasis models. Nembutal was injected intraperitoneally for anaesthesia. For the observation of the liver microcirculation, an incision was made along the edge of the right rib cage. The mouse was then placed on the stage in a lateral position with the liver being fully exposed and mounted on a cover slip. By this method, a portion of the diaphragmatic surface from the anterior lobe of the liver could be observed for 10–20 microscopic fields with a $\times$ 10 objective lens. To observe the cancer cells entering the liver microcirculation, a plastic tube was canulated into the mesenteric vein. C26 cells at a dose of $1 \times 10^6$ in 0.15 ml PBS were injected slowly for 3 minutes. The liver microcirculation was observed in this intravital microscopic system. Images were captured by a CCD camera (TEC-470, Optronics, Inc, Chelmsford, MA) connected to a VCR (BR-S605B, Victor Co, Ltd, Japan), and recorded during the cancer cell inoculation. In another group, after cancer cells injection into the ileocaecal vein, the incision was closed and the liver observation was performed after 1,3,7 and 14 days. FITC-dextran FD500S (Sigma Chemical Co, St. Louis, MO) was injected intravenously just before observation for the blood flow enhancement. EL-4 cells at $5 \times 10^5$ in 0.2 ml PBS were inoculated via the tail vein. All tumour cell inoculations were performed 30 minutes after laparotomy. To study the distributions of the cancer cells in the liver, a number of circles with a diameter of 8 cm were drawn on the TV monitor approximately in the centre of Zone 1 or Zone 3 in pictures captured by a $\times$ 20 objective lens, while each of them roughly covered an acinus. Cancer cells that had been arrested within the circles were counted and the values compared between groups.

### Antibody blocking study

To investigate the involvement of P-selectin in the cancer cells arrest, 50 µg P-selectin antibody (anti-CD62P) was injected 10 minutes before the cancer cell inoculation into 5 mice for both EL-4 and C26 groups. $5 \times 10^5$ cancer cells were injected for each mouse. Cells immobilized in the liver were counted in each microscopic field at 1 hour after inoculation. PBS was injected into 5 mice for each group as control.

### P-selectin knockout mouse study

P-selectin knockout mice (B6-129-SelP) were used for the intravital microscopic study. Normal black mice composed the control

 © 2001 Cancer Research Campaign group. EL-4 cells ($1 \times 10^5$ in 0.2 ml PBS) were injected via the tail vein. The same method was applied in 6 P-selectin knockout mice as well as normal mice to determine the number of arrested cells in the liver. In addition, 7 P-selectin knockout mice and 8 normal mice were used to establish EL-4 liver metastasis models. 2 weeks after tumour inoculation, mouse livers in both groups were harvested and weighed. The weights for the assessed livers were compared to those of the livers from cancer cell-free normal mice, as an indicator of the metastases growth.

### Statistical analysis

Student's *t*-test was used to compare data and results with a *P* value less than 0.05 were considered statistically significant.

### RESULTS

### Intravital microscopic study

The afferent portal tracts divide the liver into acini. The regions of the acinus are described as Zone 1 – periportal, Zone 2 – midlobular; and Zone 3 – centrilobular (Millward-Sadler and Jezequel, 1984). When C26 cells were inoculated into mesenteric veins, the entrapment of the cells in the liver was observed. Under the intravital microscope, the cells flowed into the liver along with the blood stream and stopped abruptly when they encountered the vessels that were much smaller in diameter than their own (Figure 1A). There were no signs of rolling before the cell arrest. On the other hand, in the lymphoma model, the sticking of the EL-4 cells could be observed on the wall of the vessels, while the blood in the lumen kept circulating (Figure 1B, C). Most of the C26 cells were arrested in the periportal (Zone 1) areas, as a consequence of the mechanical entrapment at the entrance of sinusoids. Some of the cells entered the sinusoid network, but none was found in hepatic venules (Zone 3) in the microscopic fields examined. In contrast, most of the EL-4 cells were arrested within sinusoids as a result of what appeared to be a specific interaction between the cells and the endothelium, and these cells were distributed much more evenly near Zone 1 and Zone 3 than the C26 cells (Figure 2). Some EL-4 cells passed by the hepatic venules, leaving the liver microcirculation. Once EL-4 cells were arrested in the liver, none of them was observed to detach from the endothelium and re-enter the circulation. C26 cells were observed for a 2-week period. At about 30–60 minutes after the initial stoppage, the shape of C26 cells became flat. At the third day after inoculation, some cell clusters, probably the result of the proliferation of C26 cells, could be seen in some microscopic fields (Figure 3A). At day 14, a small tumour mass could be observed under the microscope. The tumour was spherical and hypovascularized. The liver parenchyma was compressed and the vessels appeared distorted at the edge (Figure 3B).

### Immunohistochemistry and flow cytometry

Immunostaining in the liver sections from the C57BL/6 mouse showed that P-selectin was expressed as early as 30 minutes after laparotomy. However, no P-selectin expression could be detected in liver sections from P-selectin knockout mice following laparotomy (not shown). The sites of expression included the endothelium of sinusoids and venules. Flow cytometry studies using monoclonal antibodies confirmed that EL-4 cells expressed *in*

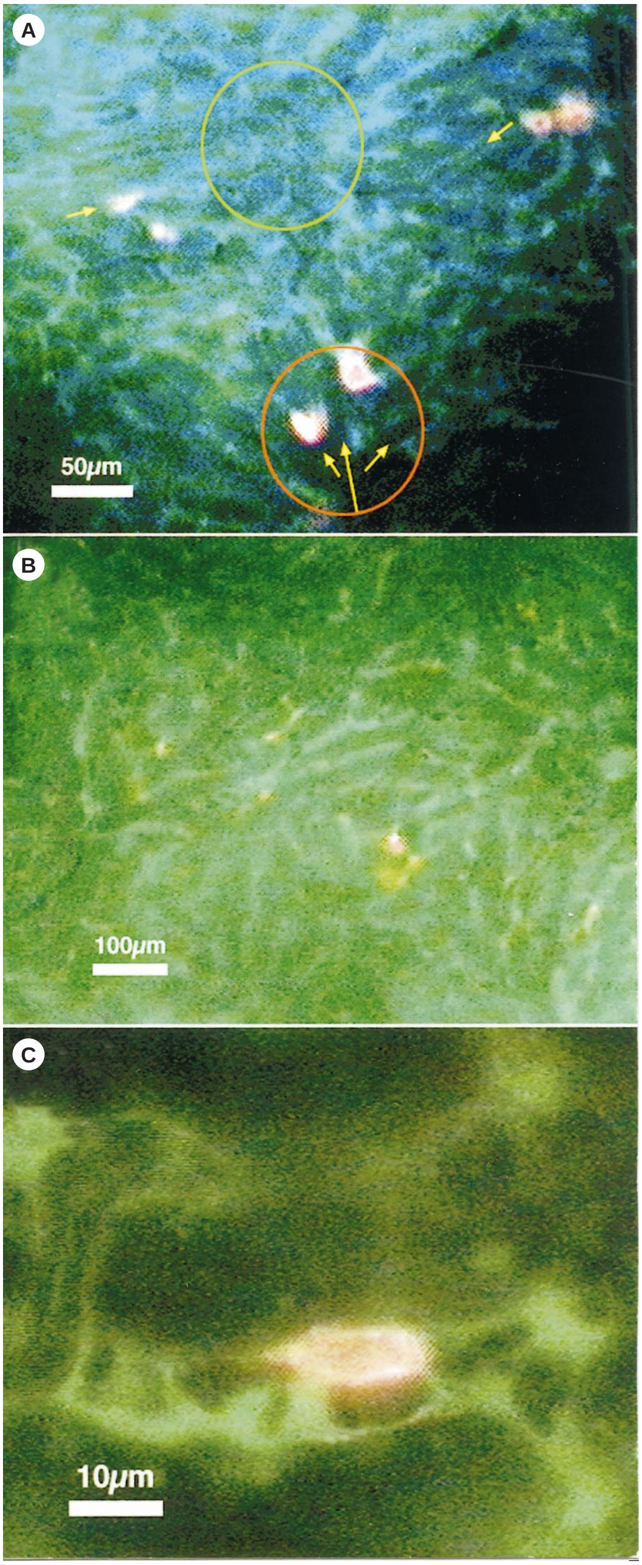

**Figure 1** Fluorescence-labelled cancer cell arrest in the liver microcirculation. (**A**) C26 cells were trapped at the entrance of the sinusoids and the vessels were obstructed. The red circle denotes the periportal venule area (Zone 1), while the green circle denotes the perihepatic venule area (Zone 3). As shown in this figure, no C26 cells reached Zone 3 areas. The arrows indicate the blood flow direction. (**B**) EL-4 cells have reached the sinusoid network. (**C**) At a higher magnification, it could be observed that EL-4 cells adhered to the sinusoid wall and the red blood cell traffic has not been interrupted

© 2001 Cancer Research Campaign

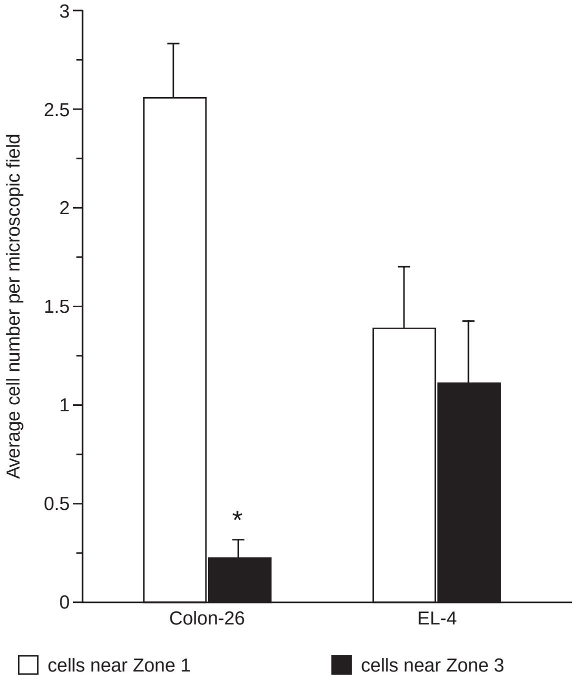

**Figure 2** Distribution of cancer cells in the liver immediately after injection via the mesenteric vein. C26 cells are predominantly located in areas near Zone 1. The number of EL-4 cells near Zone 1 was not significantly different from that near Zone 3, whereas there was a significant difference between the number of C26 cells near Zones 1 and 3, respectively (**P* < 0.001)

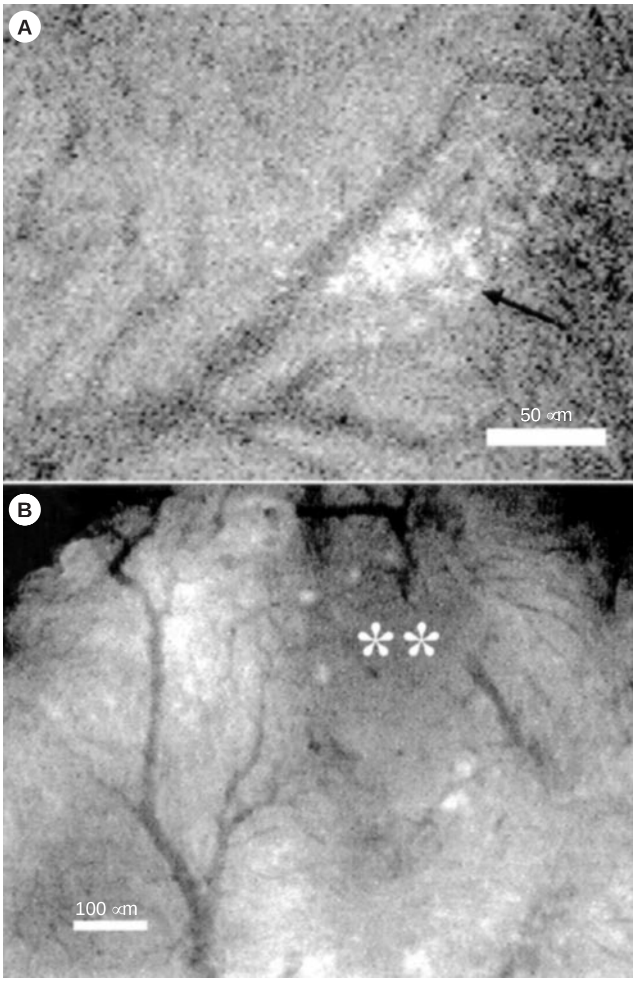

**Figure 3** Observation of the multistage process of metastasis formation. (**A**), at day 3 after cancer cell inoculation, a colony of C26 cells was observed near portal venules. 2 weeks after inoculation, the dark area marked with ** (**B**) was confirmed histologically to be a metastatic tumour of C26. It appeared as a hypovascularized tumour, but vessel sprouts could be observed, prefiguring the tumour angiogenesis

*vitro* both $sLe^x$ and $sLe^a$. Moreover, P-selectin and E-selectin were also expressed on the surface of this cancer cell line (Figure 4A). C26 cells expressed E-selectin (Figure 4B), but not P-selectin or their ligands (not shown).

### Anti-P-selectin antibody blocking experiment

There was no significant difference in the number of C26 cells trapped in the liver between anti-P-selectin mAb (anti-CD62P) pre-treated mice and control mice. In sharp contrast, when mice were injected with anti-CD62P, the number of EL-4 cells arrested in the liver diminished significantly as compared to the control group (Figure 5).

### P-selectin knockout mice study

Finally, our results demonstrated that after EL-4 cell inoculation, the number of cells arrested in the liver was significantly reduced in P-selectin knockout mice as compared to normal mice (Figure 6). Although the inoculation of EL-4 cells resulted in liver metastases at day 14 in all mice from P-selectin knockout group as well as wild-type group, the weight of the livers showed a significant difference in the total metastastatic burden between the two groups (Figure 7).

### DISCUSSION

The application of the methods described above allowed the confirmation of previous mechanisms described for the factors involved in the metastasis processes, while also intending to shed some light on the different features of the liver metastasis of a solid tumour compared to a lymphoma. In particular, the arrest process of circulating tumour cells in the liver microcirculation was directly observed and recorded through the intravital microscopic system. The arrest of C26 cells did not appear to be the result of an interaction with the endothelium by adhesive reactions. Instead, they were trapped promptly at the narrow entrance of sinusoids (Zone 1) without decelerating or rolling in advance. This finding is consistent with those reported by other groups who conducted similar intravital microscopic studies (Barbera-Guillem et al, 1989; Morris et al, 1993). Kawakami et al (1994) reported the $sLe^x$ presence at the protein level in C26 metastases in liver, suggesting a central role for this selectin ligand. In our study, we observed that the C26 cell did not express in vitro the selectin ligands $sLe^a$ or $sLe^x$. Moreover, some studies confirmed the reduced importance of selectins in metastasis for this cell line (Rodolfo et al, 1994). In our setting, the P-selectin blocking by a monoclonal anti-CD62P failed to reduce the number of C26 cells arrested in the liver. Taken together, our results emphasize that it is the physical entrapment caused by the difference in size between the solid tumour cells and the sinusoids that is the predominant mechanism for the arrest of circulating C26 cells, the initial step of a series of events resulting in liver metastasis. A characteristic feature of the solid

 © 2001 Cancer Research Campaign

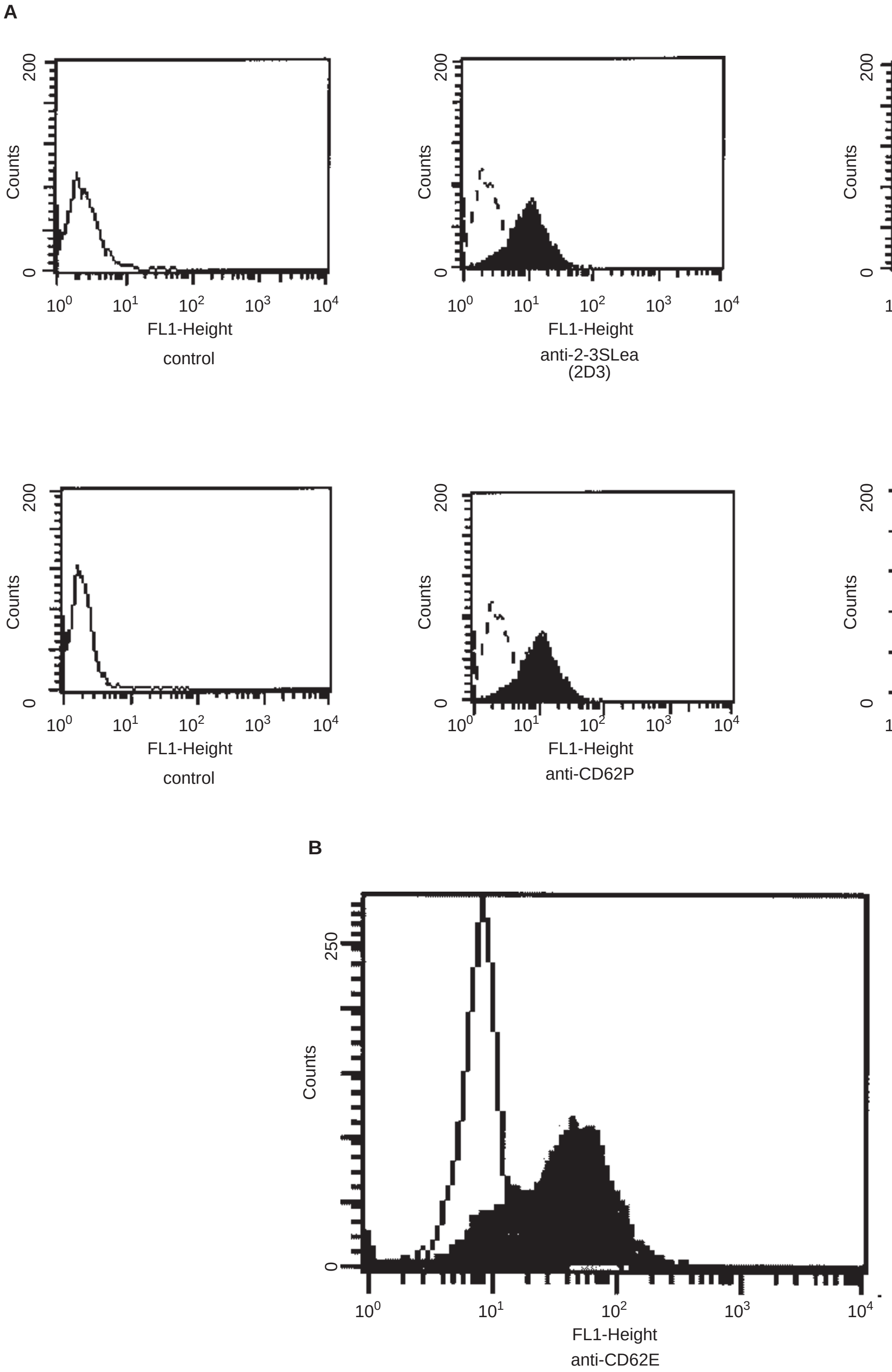

**Figure 4** Flow cytometry data, after 30 minutes incubation of tumour cells with monoclonal antibodies for CD62P, CD62E, 2D3 and 2H5. It is demonstrated that EL-4 lymphoma cells express both $sLe^x$ and $sLe^a$, as well as P-selectin and E-selectin (**A**). Dashed lines represent the control. In contrast, colon adenocarcinoma C26 cells express just E-selectin (**B**)

tumours is that the size of the cell is much bigger than the diameter of the sinusoid (25 μm vs. 6 μm in the case of C26). Moreover, the blood flow in the liver is intermittent and the shear force is relatively weak (MacPhee et al, 1995). All these factors may contribute to the entrapment of C26 cells. Our result explains the clinical feature that colon cancers always develop their first haematogenous metastasis in the liver. Cancer cells are able to deform themselves in order to pass through some narrow structures of the body (Hosaka et al, 1979; Khato et al, 1979). The deformation of C26 cells was not observed immediately after their arrival at the entrance of the sinusoid in the present study. The shape of C26 cell was increasingly flattened against the vascular wall

*© 2001 Cancer Research Campaign*

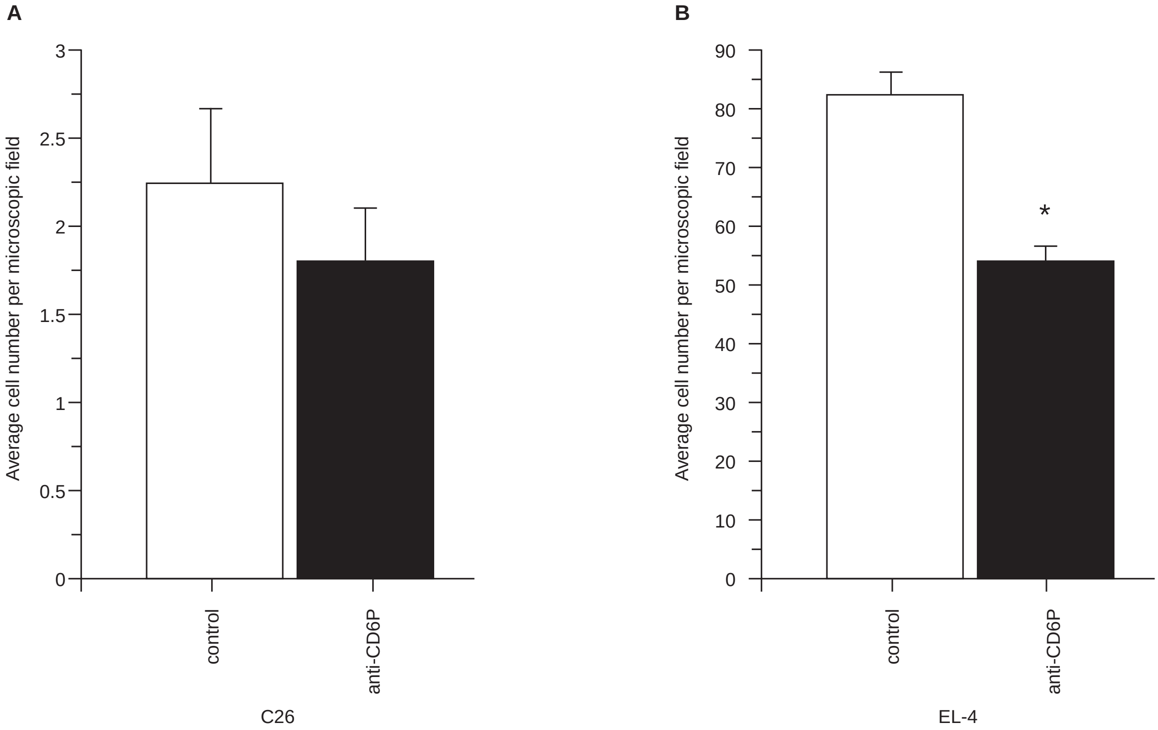

**Figure 5** The inhibitory effect after administration of P-selectin antibody on the cancer cell arrest. The ordinates indicate the average number of cancer cells arrested in the liver per microscopic field, counted using × 10 and × 20 objective lenses: (**A**) in C26 models, although there was a reduction of cell numbers in anti-P-selectin antibody pre-treated mice, it did not reach the level of significance. In contrast, the anti-P-selectin antibody significantly suppressed the arrest of EL-4 cells in the liver (**B**) as compared to the control group (* $P < 0.0001$).

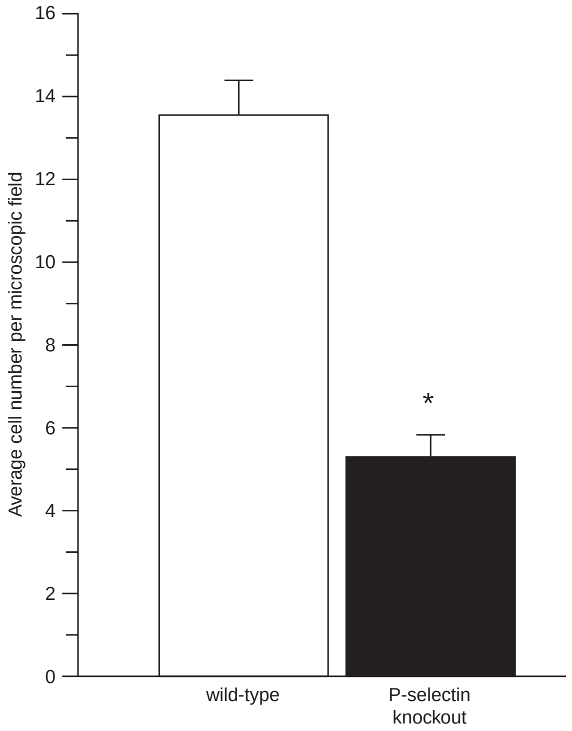

**Figure 6** The arrest of EL-4 cells in P-selectin knockout mice and in normal mice. The number of cells arrested was significantly reduced in P-selectin knockout mice (*$P < 0.0001$)

30–60 minutes after the immobilization. However, the entrapped cells remained at their original sites without further advancing into the sinusoids as late as 6 hours after observation. This suggested that an adhesive event had occurred during this interval, resulting in the stable arrest of the cancer cells on the endothelium. Some adhesion molecules like integrins, members of Ig-superfamily may contribute to this effect after the initial halt of cancer cells, and probably also facilitate cell extravasation, another essential step for tumour metastasis formation. In addition, a previous study in our group demonstrated that C26 cells inoculated via the tail vein promoted metastases in the lungs, and that a metalloproteinase inhibitor controlled their formation (Lozonschi et al, 1999). The observations in the present experiment also confirmed the fact that cancer cell mitoses could only be found outside the blood vessels, as had been reported by others (Morris et al, 1993).

Although the physical entrapment theory is confirmed by our results, it cannot explain all cancer metastasis phenomena. For example, prostate cancer always has its highest metastatic rate in bone tissues rather than in the lung, whereas cutaneous malignant melanomas preferentially metastasize to the brain. Therefore, a major role in the initiation of metastasis of some tumours is probably played by molecules involved in specific adhesion events.

EL-4 lymphoma cells were able to pass through the liver sinusoids due to their size, which is comparable to that of large lymphocytes 12–15 µm. In a preliminary experiment, all of the 10 mice inoculated with EL-4 cells via tail vein injection developed liver metastases, in 2 there were found concomitant kidney metastases, and in none of them were detected macroscopic lung metas-

 © 2001 Cancer Research Campaign

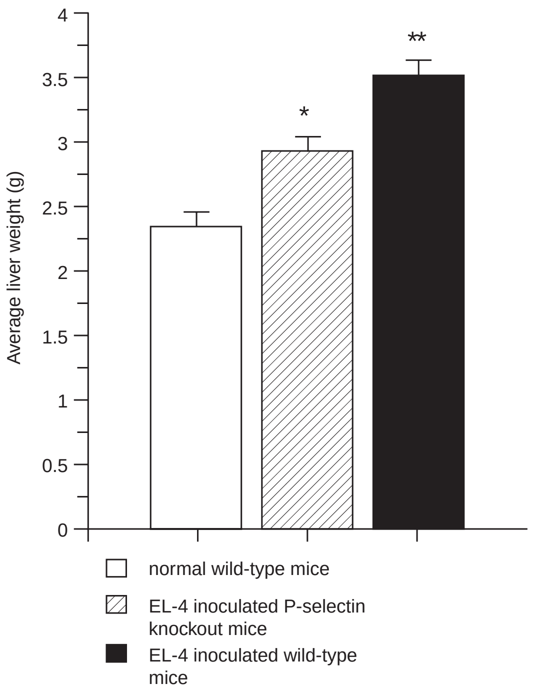

**Figure 7** Average liver weight for normal mice not challenged with tumour cells, and also for normal mice and P-selectin knockout mice 2 weeks after EL-4 inoculation. In inoculated normal mice the average liver weight was significantly higher (** $P < 0.01$) than in inoculated P-selectin knockout mice, indicating a lower metastatic burden in the knockouts than in normal mice tases. Portal vein inoculation was also carried out and identical liver metastasis ensued. To avoid frequent laparotomy, tail vein injection was chosen as the EL-4 inoculation route. The flux of EL-4 cells into the liver could be observed in the first 3–5 minutes after inoculation. The detachment and re-circulation of some arrested cells also occurred. This resulted in a varying number of arrested cells in the early period after inoculation. We have carried out a preliminary study that showed that the number of arrested cells became stable 1 hour after injection. Therefore, the counting of the arrested EL-4 cells was performed 1 hour after inoculation.

P-selectin is stored in the Weibel-Palade body in vascular endothelial cells and platelets (Bevilacqua and Nelson, 1993). Many substances (histamine, $H_2O_2$, etc.) can stimulate the expression of P-selectin molecules on the cell surface. In addition, tumour necrosis factor-alpha (TNF-α) and lipopolysaccharide were reported to trigger the de novo P-selectin biosynthesis, acting at the transcriptional level, and result in P-selectin overexpression (Gotsch et al, 1994). Unlike the transient expression induced by histamine or $H_2O_2$, cytokines induced a prolonged P-selectin expression. TNF-α has been shown to have a high serum level in cancer patients (Ardizoia et al, 1992). Consequently, it should be considered that P-selectin is expressed on the endothelial cells in cancer-bearing individuals.

All tumour cell inoculations were performed at 30 minutes after laparotomy, at which time point P-selectin could be detected by immunohistochemical staining. The administration of P-selectin antibody partially blocked the arrest of EL-4 cells. Also, the number of EL-4 cells arrested was significantly reduced in P-selectin knockout mice as compared with normal mice. The comparison between the weight of the livers from tumour challenged mice to that of untreated mice showed that EL-4 liver metastasis was suppressed in P-selectin knockout mice as compared to normal mice. However, although both the administration of neutralizing monoclonal antibody for P-selectin and the use of P-selectin knockout mice resulted in a lower total metastatic burden, EL-4 cell arresting and liver metastasis could not be completely inhibited in any of these settings. Many other adhesion molecules such as E-selectin, integrins or Ig-superfamily molecules may also have their roles in this process. We aim to investigate the involvement of other adhesion molecules as well as the carbohydrate ligands of selectins in further studies.

It remains unclear why EL-4 cells do not form metastases in the lung but do so specifically in the liver. P-selectin does not seem to play the determinant role, because it could also be detected in the lung by immunohistochemistry in our animal model (not shown). Moreover, immobilized EL-4 cells were also detected at the lung specimen examination after their tail vein inoculation. These results prompted the hypothesis that some additional signals are necessary after the initial arrest to affect another phase of metastatic process such as cell extravasation and migration, angiogenesis or cell proliferation.

The direct observation of the behaviour of circulating tumour cells in our study indicates 2 distinct mechanisms that contribute to the initial arrest of circulating cancer cells in a target organ for metastasis – the liver. One is the mechanical entrapment of solid cancer cells in the microcirculation, while the other involves the preferential adhesion of cancer cells to the endothelium. P-selectin has been demonstrated to play a significant role in the latter mechanism.

### ACKNOWLEDGEMENTS

We thank Emiko Shibuya and Hiroko Fujimura for their excellent technical assistance. This work was supported by a grant (05807094) from the Ministry of Education, Sports, Science, and Culture, Japan.

### REFERENCES

Aigner S, Ramos CL, Hafezi-Moghadam A, Lawrence MB, Friederichs J, Altevogt P and Ley K (1998) CD24 mediates rolling of breast carcinoma cells on P-selectin. *FASEB J* **12**: 1241–1251

Ardizzoia A, Lissoni P, Brivio F, Tisi E, Perego MS and Grassi MG (1992) Tumor necrosis factor in solid tumors: increased blood levels in the metastatic disease. *J Biol Regul Homeost Agents* **6**: 103–107

Barbera-Guillem E, Alonso-Varona A and Vidal-Vanaclocha F (1989) Selective implantation and growth in rats and mice of experimental liver metastasis in acinar zone one. *Cancer Res* **49**: 4003–4010

Bevilacqua MP and Nelson RM (1993) Selectins. *J Clin Invest* **91**: 379–387

Biancone L, Araki M, Araki K, Vassali P and Stamenkovici (1996) Redirection of tumor metastasis by expression of E-selectin in vivo. *J Exp Med* **183**: 581–587

Chambers AF (1999) The metastatic process: basic research and clinical implications. *Oncol Res* **11**: 161–168

Chambers AF, MacDonald IC, Schmidt EE, Koop S, Morris VL, Khokha R and Groom AC (1995) Steps in tumor metastasis: new concepts from intravital videomicroscopy. *Cancer Metastasis Rev* **14**: 279–301

Fox-Robichaud A and Kubes P (2000) Molecular mechanisms of tumor necrosis factor a-stimulated leukocyte recruitment into the murine hepatic circulation. *Hepathology* **31**: 1123–1127

Gotsch U, Jager U, Dominis M and Vestweber D (1994) Expression of P-selectin on endothelial cells is upregulated by LPS and TNF-alpha in vivo. *Cell Adhesion & Communication* **2**: 7–14

Horan PK and Slezak SE (1989) Stable cell membrane labeling. *Nature* **340**: 167–168

Hosaka S, Suzuki M and Sato H (1979) Leucocyte-like motility of cancer cells, with reference to the mechanism of extravasation in metastasis. *Gann* **70**: 559–561

Izumi Y, Taniuchi Y, Tsuji T, Smith CW, Nakamori S, Fidler IJ and Irimura T (1995) Characterization of human colon carcinoma variant cells selected for sialyl Lex carbohydrate antigen: liver colonization and adhesion to vascular endothelial cells. *Exp Cell Res* **216**: 215–221

Kan Z, Ivancev K, Lunderquist A, McCuskey PA, Wright KC, Wallace S and McCuskey RS (1993) In vivo microscopy of hepatic tumors in animal models: a dynamic investigation of blood supply to hepatic metastasis. *Radiology* **187**: 621–626

Kawakami H, Ito M, Miura Y and Hirano H (1994) Expression of Lewis(x) sugar structure in the liver metastasis of mouse colon carcinoma (colon 26) cells. *Clin Exp Metastasis* **12**: 129–133

Khato J, Sato H, Suzuki M and Sato H (1979) Filterability and flow characteristics of leukemic and non-leukemic tumor cell suspension through polycarbonate filters in relation to hematogenous spread of cancer. *Tohoku J Exp Med* **128**: 273–284

Lauri D, Needham L, Martin-Padura I and Dejana E (1991) Tumor cell adhesion to endothelial cells: endothelial leukocyte adhesion molecule-1 as an inducible adhesive receptor specific for colon carcinoma cells. *J Natl Cancer Inst* **83**: 1321–1324

Lozonschi L, Sunamura M, Kobari M, Egawa S, Ding L and Matsuno S (1999) Controlling tumor angiogenesis and metastasis of C26 murine colon adenocarcinoma by a new matrix metalloproteinase inhibitor, KB-R7785, in two tumor models. *Cancer Res* **59**: 1252–1258

MacPhee PJ, Schmidt EE and Groom AC (1995) Intermittence of blood flow in liver sinusoids, studied by high-resolution in vivo microscopy. *Am J Physiol* **269**: G692–G698

Millward-Sadler GH and Jezequel AM (1984) Normal histology and ultrastructure. In Liver and Biliary Disease, pp. 13–15 W.B. Sanders: Philadelphia

Morris VL, MacDonald IC, Koop S, Schmidt EE, Chambers AF and Groom AC (1993) Early interactions of cancer cells with the microvasculature in mouse liver and muscle during hematogenous metastasis: videomicroscopic analysis. *Clin Exp Metastasis* **11**: 377–390

Nakamori S, Kameyama M, Imaoka S, Furukawa H, Ishikawa O, Sasaki Y, Kabuto T, Iwanaga T, Matsushita Y and Irimura T (1993) Increased expression of sialyl Lewis X antigen correlates with poor survival in patients with colorectal carcinoma: clinicopathological and immunohistochemical study. *Cancer Res* **53**: 3632–3637

Nicolson G (1988) Organ specificity of tumor metastasis: role of preferential adhesion, invasion and growth of malignant cells at specific secondary sites. *Cancer Metastasis Rev* **7**: 143–188

Orr FW, Wang HH, Lafrenie RM, Scherbarth S and Nance DM (2000) Interactions between cancer cells and the endothelium in metastasis. *J Pathol* **190**: 310–329

Patel JK, Didolkar MS, Pickren JW and Moore RH (1978) Metastatic pattern of malignant melanoma. A study of 216 autopsy cases. *Am J Surg* **135**: 807–810

Pauli BU, Augustin-Voss HG, El-Sabban ME, Johnson RC and Hammer DA (1990) Organ-preferences of metastasis. The role of endothelial cell adhesion molecules. *Cancer Metastasis Rev* **9**: 175–189

Rodolfo M, Castelli C, Bassi C, Accornero P, Sensi M and Parmiani G (1994) Cytotoxic T lymphocytes recognize tumor antigens of a murine colonic carcinoma by using different T-cell receptors. *Int J Cancer* **57**: 440–447

Scherbarth S and Orr FW (1997) Intravital videomicroscopic evidence for regulation of metastasis by the hepatic microvasculature: effects of interleukin-1alpha on metastasis and the location of B16F1 melanoma cell arrest. *Cancer Res* **57**: 4105–4110

Sugarbaker EV (1981) Patterns of metastasis in human malignancies. *Cancer Biol Rev* **2**: 235–278

Tarin D and Matsumira Y (1994) Recent advances in the study of tumor invasion and metastasis. *J Clin Pathol* **47**: 385–390

Wang HH, Nance DM and Orr FW (1999) Murine hepatic microvascular adhesion molecule expression is inducible and has a zonal distribution. *Clin Exp Metastasis* **17**: 149–155

Zetter BR (1993) Adhesion molecules in tumor metastasis. *Semin Cancer Biol* **4**: 219–229

Zhu DZ, Cheng CF and Pauli BU (1991) Mediation of lung metastasis of murine melanomas by a lung-specific endothelial cell adhesion molecule. *Proc Natl Acad Sci USA* **88**: 9568–9572

 *© 2001 Cancer Research Campaign*
